# Mean pulmonary artery pressure prediction with explainable multi-view cardiovascular magnetic resonance cine series deep learning model

**DOI:** 10.1016/j.jocmr.2024.101133

**Published:** 2024-12-05

**Authors:** Li-Hsin Cheng, Xiaowu Sun, Charlie Elliot, Robin Condliffe, David G. Kiely, Samer Alabed, Andrew J. Swift, Rob J. van der Geest, the ASPIRE Consortium, David G Kiely, Lisa Watson, Iain Armstrong, Catherine Billings, Athanasios Charalampopoulos, Robin Condliffe, Charlie Elliot, Abdul Hameed, Neil Hamilton, Judith Hurdman, Allan Lawrie, Robert A Lewis, Smitha Rajaram, Alex Rothman, Andy J. Swift, Steven Wood, AA Roger Thompson, Jim Wild

**Affiliations:** e Sheffield Pulmonary Vascular Disease Unit, Sheffield Teaching Hospitals NHS Foundation Trust, Royal Hallamshire Hospital, Sheffield UK; f National Institute for Health and Care Research (NIHR) Sheffield Biomedical Research Centre (NIHR203321); g University of Sheffield, Sheffield, UK; a Division of Image Processing (LKEB), Department of Radiology, Leiden University Medical, Center, the Netherlands; b Sheffield Pulmonary Vascular Disease Unit, Sheffield Teaching Hospitals NHS Foundation, Trust, UK; c Department of Infection, Immunity and Cardiovascular Disease, University of Sheffield, UK; d National Institute for Health and Care Research (NIHR) Sheffield Biomedical Research Centre, Sheffield, UK

**Keywords:** Mean pulmonary artery pressure, Pulmonary hypertension, Multi-view cardiac MR, Deep learning, Explainable AI

## Abstract

**Background:**

Pulmonary hypertension (PH) is a heterogeneous condition and regardless of etiology impacts negatively on survival. Diagnosis of PH is based on hemodynamic parameters measured invasively at right heart catheterization (RHC); however, a non-invasive alternative would be clinically valuable. Our aim was to estimate RHC parameters non-invasively from cardiac magnetic resonance (MR) data using deep learning models and to identify key contributing imaging features.

**Methods:**

We constructed an explainable convolutional neural network (CNN) taking cardiac MR cine series from four different views as input to predict mean pulmonary artery pressure (mPAP). The model was trained and evaluated on 1646 examinations. The model’s attention weight and predictive performance associated with each frame, view, or phase were used to judge its importance. Additionally, the importance of each cardiac chamber was inferred by perturbing part of the input pixels.

**Results:**

The model achieved a Pearson correlation coefficient of 0.80 and R^2^ of 0.64 in predicting mPAP and identified the right ventricle region on short-axis view to be especially informative.

**Conclusion:**

Hemodynamic parameters can be estimated non-invasively with a CNN, using MR cine series from four views, revealing key contributing features at the same time.

## Graphical abstract

**Figure:**
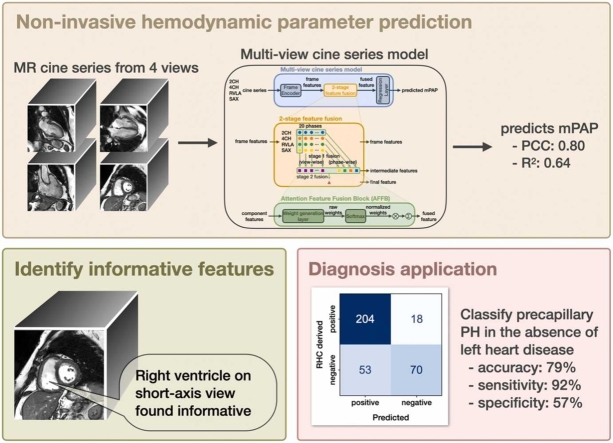

## 1 Introduction

Pulmonary hypertension (PH) is a hemodynamic state characterized by an elevation of blood pressure in the pulmonary arteries. It is classified into five major groups [1]. Forms of PH where treatments directed at the pulmonary vasculature result in improved outcomes include pulmonary arterial hypertension (PAH) (group 1), chronic thromboembolic PH (group 4), and more recently demonstrated, some forms of, PH in association with interstitial lung disease (group 3) [1]. Forms of PH that are either untreatable or where therapy options are unclear include PH associated with left heart disease (LHD) (group 2), associated with lung disease and or hypoxia (group 3 with the exception of interstitial lung disease), and in addition PAH with unclear and/or multifactorial mechanisms (group 5). In all instances, PH is a life-shortening condition [1], [2]. Thus, both accurate diagnosis of PH and subgroup phenotyping are essential for better patient outcomes. Echocardiography is often the first-line test raising the suspicion of PH, whereas a definite diagnosis is based on hemodynamic parameters measured by right heart catheterization (RHC). During RHC, a catheter is inserted into the pulmonary artery to measure the mean pulmonary artery pressure (mPAP). A mPAP greater than 20 mmHg confirms the diagnosis of PH [1]. RHC is a procedure with small but significant risks. Due to the invasive nature of the procedure and the necessity of specialized operators, it is on some occasions not feasible [3], [4]. A non-invasive estimation of the hemodynamic parameters can therefore be clinically valuable.

Imaging data provides evidence of the pathophysiological manifestations of PH, and may provide the basis for a non-invasive alternative method to estimate mPAP [5], [6]. Both Doppler estimation of pulmonary artery systolic pressure and signs of right ventricle (RV) dysfunction or dilatation with two-dimensional (2D) echocardiography can raise the suspicion of PH as well as the cause [5], [7]. In addition, cardiovascular magnetic resonance (CMR) enables comprehensive cardiac evaluation, including the assessment of morphology and function, and has prognostic value in PAH [5], [8], [9]. However, visual assessment of the shape and structure of the heart can be time-consuming, expertise-demanding, and subjective. Therefore, an image–based non-invasive alternative to RHC that was automatic, reproducible, and quantitative would be highly desirable.

There are reports that adopt a machine learning [10], [11], [12], [13], [14], [15] or deep learning [16], [17] approach and satisfy the automatic, reproducible, and quantitative requirements. Regarding the modality and view, there are studies using short-axis (SAX) cine CMR [10], [11], [12], long-axis four-chamber (4CH) cine CMR [13], the two views separately [14], or the two views together [15]. Other studies [16], [17] fuse multiple views from echocardiography. Regarding the feature and automation, there are methods based on predefined features, such as interventricular septal angle, ventricular mass index, or right atrial (RA) area [10], [11], [12], [13], which demand image segmentation and thus manual annotation for at least training. Other approaches allow the model to identify useful features freely [14], [15], [16], [17], requiring no manual annotation. Finally, regarding the prediction target, most of the studies focus on classification tasks, such as identifying PH [10], [17], PAH [14], [16], precapillary PH [11], or elevated pulmonary artery wedge pressure (PAWP) [15]. Others have evaluated the correlation between predefined features with hemodynamic parameters [10], [12], [13]. Of the models that use learned features, some provide explainability which allows users to understand partially how the model reaches certain conclusions [14], [17]. In this work, we sought to combine the strengths of previous works to develop a convolutional neural network (CNN), taking MRI cine series of four different views as input and regressing mPAP directly. Additionally, we sought to inspect the key features considered by the model to be especially informative, revealing important views, cardiac phases, and cardiac chambers.

## 2 Methods

### 2.1 Patients and metadata

Consecutive incident patients suspected of having PH who were referred to a tertiary PH center for CMR from 2007 to 2021 were identified from the Assessing the Spectrum of Pulmonary Hypertension Identified at a Referral Center (ASPIRE) database [18]. Examinations fulfilling the following criteria were included: no missing data in MR image and RHC label; CMR acquired by General Electic machines; CMR and RHC performed within 48 h. This results in 1646 examinations in total, consisting of 1535 unique patients. The patients were randomly split into training, validation, and testing sets, with the ratio of 65%, 15%, 20%, respectively. An overview of the data across training, validation, and test sets is presented in Table 1.

**Table 1:** Data distribution.

	Training set	Validation set	Testing set
Number of patients	974	238	323
Sex female (n, %)	610, 63%	150, 63%	208, 64%
Number of examinations	1041	260	345
Age (years)	63.7 ± 14.1	63.2 ± 14.2	64.0 ± 13.5
WHO functional class I/II/III/IV/missing (n)	4/101/828/88/20	1/15/218/21/5	3/37/275/24/6
mPAP (mmHg)	39.7 ± 14.5	39.8 ± 13.4	39.9 ± 14.1
PAWP (mmHg)	12.9 ± 5.4	12.7 ± 5.8	12.7 ± 5.8
PVR (Wood units)	6.3 ± 5.0	6.3 ± 4.6	6.5 ± 5.0

*mPAP* mean pulmonary artery pressure, *PAWP* pulmonary artery wedge pressure, *PVR* pulmonary vascular resistance, *WHO* World Health Organization

RHC was performed as part of the routine clinical pathway, using a balloon-tip 7.5-F thermodilution catheter (Becton-Dickinson, Franklin Lakes, New Jersey). For this study, PH was defined as a resting mPAP greater than 20 mmHg.

### 2.2 MR image acquisition and processing

CMR was performed using a 1.5T whole-body imager (HDx; GE Healthcare, Milwaukee, Wisconsin), with the patient supine, using an eight-channel cardiac coil. Standard two-chamber (2CH), 4CH, and right ventricular long-axis (RVLA) balanced steady-state free precession cine images were acquired. In addition, an SAX stack was acquired from base to apex. For each view, the cine series consists of 20 phases. For the SAX view, the middle slice is taken as a representative slice of the stack. As a result, in this work, all four views are 2D cine series.

**Fig. 1:**
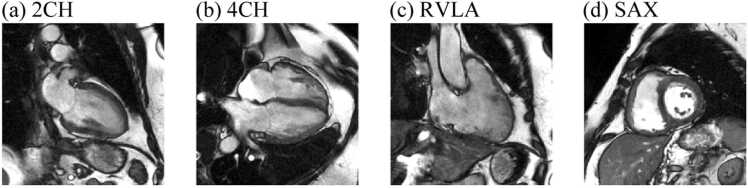
An example of the processed CMR cine series at phase 1. (a)-(d) 2CH, 4CH, RVLA, and SAX views, respectivley. *2CH* two-chamber, *4CH* four-chamber, *CMR* cardiovascular magnetic resonance, *RVLA* right ventricular long-axis, *SAX* short axis

For each cine series, we first performed intensity normalization by clipping the intensity at the 3rd and 97th percentile, then normalize the intensity to 0 to 1. Next, all images were scaled to the same pixel spacing of 0.94 mm per pixel. Finally, images were cropped into a matrix size of 256 × 256 pixels. The field of view was thus 240 × 240 mm^2^. Fig. 1 shows an example of the processed images. During training, on-the-fly image augmentations were applied to each cine series, including random flipping, shifting, scaling, and rotation.

### 2.3 Multi-view cine series deep learning model

In essence, the multi-view cine series model received cine series from four views as input and predicted mPAP as output. The frame features were fused at each phase or view and then into the final feature. Within the process, frames, views, or phases are all “features,” and were evaluated based on both their predictive performance and attention weights, giving insights into which component is the key contributing feature for the prediction.

**Fig. 2:**
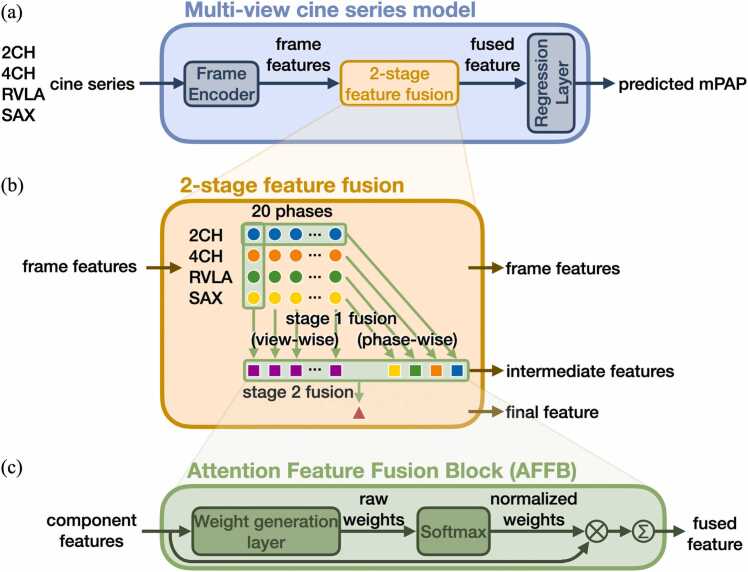
Multi-view cine series model. (a) The model uses cine series from multiple views to generate a regression prediction. The input cine series are encoded into frame features, fused, and then processed by the Regression Layer to predict the mPAP. (b) The two-stage feature fusion strategy fuses frame features into intermediate features then into the final feature. (c) The Attention Feature Fusion Block (AFFB) is the key component in two-stage feature fusion. It fuses an arbitrary number of input features into one output feature of the same dimension by weighted-sum. *2CH* two-chamber, *4CH* four-chamber, *mPAP* mean pulmonary artery pressure, *RVLA* right ventricular long-axis, *SAX* short axis

The multi-view cine series model is shown in Fig. 2a. The shared Frame Encoder, a ResNet50 model [19], encodes each individual frame from the cine series into a feature vector. The two-stage feature fusion block, empowered by the shared Attention Feature Fusion Block (AFFB), fuses the features. Finally, the shared Regression Layer can transform either a component feature or a fused feature into a prediction.

The AFFB (Fig. 2c) fuses an arbitrary number of component features into one fused feature. Inside the AFFB, a shared linear layer generates a raw attention weight for each component feature. A Softmax operation is applied to the raw attention weights to make them sum up to one, generating the normalized attention weights for the weighted-sum operation.

The two-stage feature fusion strategy (Fig. 2b) utilizes the AFFB to fuse 80 frame features into a final feature. In stage 1, view-wise fusion fuses four frames of a particular phase into a spatial summary for that phase, and phase-wise fusion fuses 20 frames in a view into a temporal summary for that view. In stage-2 fusion, the 20 spatial and 4 temporal summaries are fused into the final feature. Mean square error between the predicted and the RHC-measured ground-truth mPAP was used as the loss function to train the model. Details can be found in Supplementary file A.

### 2.4 Evaluation

#### 2.4.1 Evaluating regression performance

The mean absolute error (MAE), Pearson correlation coefficient (PCC), and R^2^ were used to evaluate the performance of the regression task. The R^2^ is defined as in Eq. (1),

(1) $$R^{2}=1-\frac{SS_{residual}}{SS_{total}}$$

where $SS_{residual}$ is the sum of the squares of the difference between the ground truth and prediction, and $SS_{total}$ is the sum of the squares of the difference between the ground truth and its average. The value of R^2^ can therefore be negative.

#### 2.4.2 Evaluating frame, view, and phase importance

The architecture of the model offers two types of built-in feature importance explanations. First, since the feature fusion is dimension-preserving, each individual component feature or fused feature can undergo the Regression Layer and be evaluated by its predictive performance. On the other hand, during fusion, each component feature is assigned an attention weight. The weights indicate the preference of the model over each component feature.

As a result, we will compare each feature its predictive performance and attention weight relative to that of other features of the same fusion stage, inferring its relative importance. The higher both the performance and the weight, the more important a frame, view, or phase is.

#### 2.4.3 Evaluating chamber importance

Pixel perturbation was adopted to further identify important anatomical regions, i.e. cardiac chambers, in the input cine series. Two complementary approaches were used: masking out regions in the input cine series, or pasting regions in the cine series onto a black canvas to form the new input. The resulting perturbed predictive performances then reflect the relevance of the masked or pasted region.

To derive a globally summarized and quantitative explanation, we focused on the averaged perturbed performance for each chamber, instead of deriving per-instance heatmap and visually summarized the highlighted region. We used an in-house segmentation model [12], [20] to obtain chamber masks. We then applied a Gaussian filter (standard deviation = 4 pixels, radius = 16 pixels) on the masks and thresholding the values at zero, such that the segmentation boundary was slightly extended. The dilation hides both the exact area and shape (boundary curvature) information of a chamber. Only in this way, when a chamber is masked out, none of its pixel intensity, shape, or exact area information is left behind in the image, as such the performance drop reflects the full consequence of erasing a chamber. Similarly, when a chamber is pasted, the model cannot use the extra shape or area information hinted by the mask to derive accurate predictions more easily.

### 2.5 Implementation details

The model was implemented with Pytorch. The Adam optimizer was used, with learning rate set to 10^−4^ and weight decay set to 10^−8^. The batch size was 10 and the models were trained for 1000 epochs with early stop. The model was trained on a machine with an NVIDIA RTX A6000 GPU (NVidia, Santa Clara, California).

## 3 Results

### 3.1 Hemodynamic parameters prediction

**Fig. 3:**
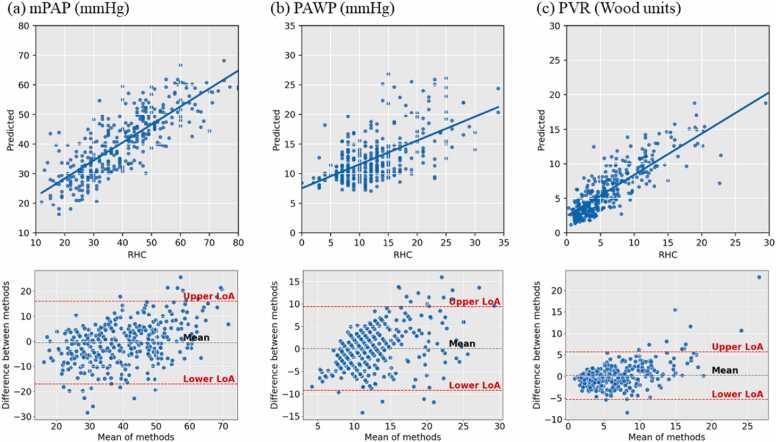
Scatter and Bland-Altman plots for predicting each hemodynamic parameter. Columns (a)-(c) are, respectively, the plots for the mPAP, PAWP, and PVR. *mPAP* mean pulmonary artery pressure, *PAWP* pulmonary artery wedge pressure, *PVR* pulmonary vascular resistance

The performances of the multi-view cine series deep learning models predicting each hemodynamic parameter are shown in Table 2 and Fig. 3, with mPAP prediction being the main focus in the following sections.

**Table 2:** Performance of predicting each hemodynamic parameter, mPAP, PAWP, and PVR.

Prediction target	mPAP	PAWP	PVR
MAE	6.5 (mmHg)	3.7 (mmHg)	1.8 (Wood units)
PCC	0.80	0.59	0.83
R^2^	0.64	0.33	0.68

*MAE* mean absolute error, *PCC* Pearson correlation coefficient, *mPAP* mean pulmonary artery pressure, *PAWP* pulmonary artery wedge pressure, *PVR* pulmonary vascular resistance

Both mPAP and PVR predictions achieved PCCs higher than 0.8 and R^2^ higher than 0.6. In contrast, for PAWP the PCC was 0.59 and the R^2^ was 0.33.

### 3.2 Diagnostic application of non-invasive RHC alternative

To further explore the application of the non-invasive hemodynamic parameter prediction models, we investigated their diagnostic potential.

**Fig. 4:**
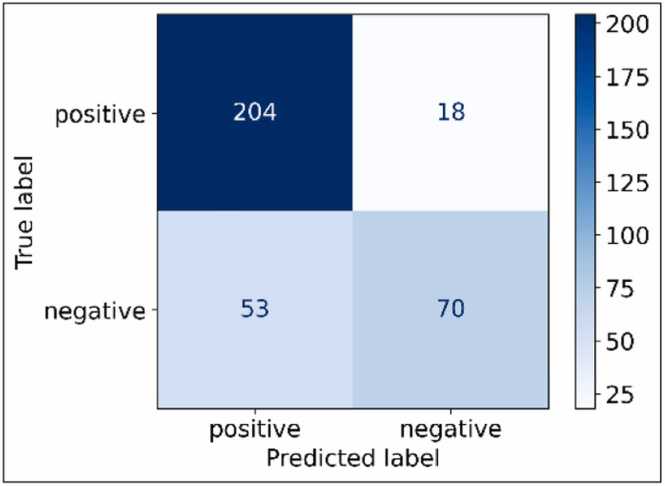
Confusion matrix of identifying precapillary PH in the absence of LHD non-invasively. *LHD* left heart disease, *PH* pulmonary hypertension

Using the three hemodynamic prediction models together, precapillary PH in the absence of LHD (mPAP > 20 mmHg, PVR > 2 Wood units, and PAWP ≤ 15 mmHg) can be identified with an accuracy of 79%, sensitivity of 92%, and specificity of 57% (Fig. 4).

### 3.3 Built-in model explainability

**Fig. 5:**
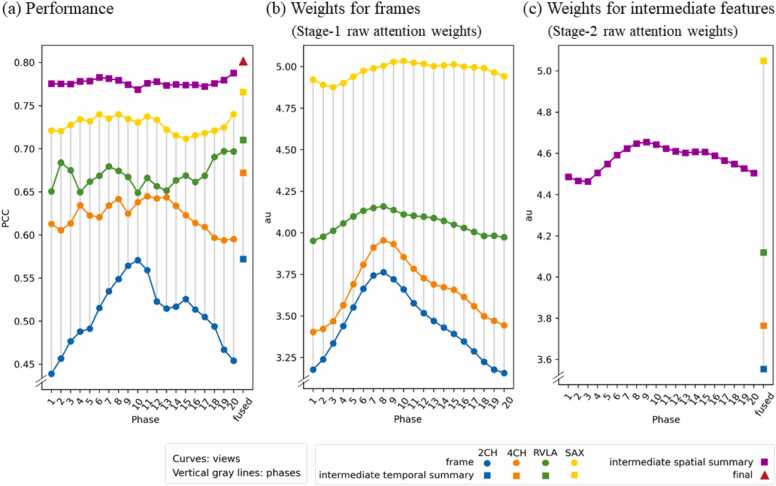
Per-feature predictive performance (a) and attention weights (b and c). The round dots represent frame features, the squares represent intermediate features, and the triangle represents the final feature. Values within each sub-plots can be compared to establish relative importance—the higher the more important a feature is. Note that the values in (b) and (c) are not cross-comparable since they are never normalized together. *2CH* two-chamber, *4CH* four-chamber, *mPAP* mean pulmonary artery pressure, *RVLA* right ventricular long-axis, *SAX* short axis

We leverage the built-in explainability of the model to reveal mPAP-relevant features, which could be a frame, a view, or a phase. The importance of each feature is evaluated both by its predictive performance (Fig. 5a) and its assigned attention weight (Fig. 5b and c).

#### 3.3.1 Influences of feature fusion

In Fig. 5a, the performances for the 80 initial frames (round dots), 24 intermediate summaries for a view or a phase (squares), and the final aggregated feature (triangle) are all presented. For any fusion, the fused feature always achieves a higher performance than any of the component features. For example, the temporal summaries are better than each of the single phases (square vs round dots of the same color); the spatial summaries are better than each of the single view (square vs round dots at the same phase); and the final feature is better than the intermediate features (triangle vs squares). This indicates that the temporal aggregation of a view is more informative than any single frame, the spatial aggregation of a phase is more informative than a single view, and the final fused feature aggregating the cine loops of all four viewpoints is the most informative.

#### 3.3.2 Informative view

The same relativity was concluded at every phase, both in terms of performance and attention weight: SAX > RVLA > 4CH > 2CH. Namely, along each vertical solid gray line in Fig. 5, the performance and weight ordering of the four views are always in this exact ordering. In this case, the component feature that is preferred by the model, which receives a higher weight, is also the feature that itself has a higher predictive performance.

SAX is concluded to be the most informative among the four views. Inspecting the normalized weights (Fig. S1a), we found that when performing view-wise fusions, the model prefers forming the fused feature with roughly 50% of the SAX component, and 50% of the mix of the other three views. Inspecting the performances, we found that when other views are not available, having only the SAX cine series achieves already a PCC of 0.77 and R^2^ of 0.58 (Fig. 5a yellow square).

#### 3.3.3 Informative phase

Unlike in the views, the performances relativity and weights relativity in the phases do not match perfectly, hence no single most-informative phase can be concluded.

Inspecting the performances, we found that the best-performing phase for each view is different (Fig. 5a). Inspecting the weights (Figs. 5b and c, S1b), we found that in fusing 2CH, 4CH, and RVLA frames, phase 8 is assigned the highest weight; while in fusing SAX frames or intermediate spatial features, phase 9 is assigned the highest weight. This corresponds to the end-systole phase.

In summary, it is observed that the model prefers to use a higher portion of end-systole components in phase-wise fusions, even though it is usually not when the component features have the highest performance.

### 3.4 Informative chamber

In addition to the built-in explainability of the model indicating informative view(s) and phase(s), the informative chamber(s) were inferred by two complementary pixel-perturbation approaches, masking and pasting.

All perturbed performances (Table 3) are to be compared to the original intact condition without perturbations, which is PCC = 0.80 as previously shown in Section 3.1. First, when masking or pasting a chamber on all presented views, RV has the most impact, dropping PCC to 0.39 in masking and preserving PCC of 0.56 in pasting (row “All views” in Table 3). Next, when masking or pasting a single chamber on a single view, RV on SAX has the most impact, dropping PCC to 0.67 in masking and preserving PCC of 0.50 in pasting (row “SAX” in Table 3). Therefore, we concluded from both masking and pasting perturbations that RV is the most informative chamber considered by the model, especially in the SAX view.

**Table 3:** The PCC resulting from (a) masking and (b) pasting perturbations.

	(a) Masking				(b) Pasting			
	LV	LA	RV	RA	LV	LA	RV	RA
2CH	0.77	0.78			0.16	−0.08		
4CH	0.78	0.79	0.77	0.78	−0.22	−0.12	0.42	0.15
RVLA			0.77	0.77			0.29	0.19
SAX	0.73		**0.67**		0.14		**0.50**	
All views	0.61	0.77	**0.39**	0.75	0.06	−0.13	**0.56**	0.22

An empty cell implies the chamber is not presented in that view. The row “All views” implies masking or pasting a chamber region in all of the views that it is presented in. (a) In masking a chamber from the input cine series, a larger performance drop (low PCC) indicates a more important region. The largest performance drop when masking a chamber on a single view or on all views were marked in bold. (b) When pasting a chamber onto a black canvas, a better-preserved performance (high PCC) indicates a more important region. The best-preserved performance when pasting a chamber on a single view or on all views were marked in bold.

*2CH* two-chamber, *4CH* four-chamber, *LA* left atrium, *LV* left ventricle, *PCC* Pearson correlation coefficient, *RA* right atrium, *RV* right ventricle, *RVLA* right ventricular long-axis, *SAX* short axis

## 4 Discussion

In this work, we have built a deep learning model to estimate pulmonary hemodynamic parameters non-invasively, demonstrated its clinical utility potential, and inspected the key features used by the model. Using four views of MRI cine series as input and without the need of segmentation, the trained CNN was able to predict mPAP with a PCC of 0.80 and R^2^ of 0.64. The predicted hemodynamic parameters were able to classify precapillary PH in the absence of LHD with an accuracy of 79%. Enabled by the built-in explainability of the model, we observed that using multiple views outperforms single views, and among the views, SAX is at the same time the most predictive and the most preferred component by the model during fusion. We also observed that using the whole cine series as model input outperforms steady frames. The model prefers end-systole components during phase-wise fusion, although there is no obvious consensus among the views on which single phase has the best performance. Finally, by masking and pasting part of the images, we observed that the model depends highly on the RV region, especially in the SAX view.

Of note, the current model does not always assign the highest attention weight to the best-performing component feature. For example, the performances and weights for the phases do not match perfectly, making it inconclusive which component is the most informative. We have several possible explanations for this. First, it is possible that components that individually do not have the highest performance are the best choice for forming the fused feature. Weights should probably be interpreted as the recipe to generate a good fusion result, which will not always correspond to the performance of individual component features. Second, the end-systole components might be more robust, such that the model prefers to rely on them. Third, the weight-generating linear layer within the AFFB might not be good enough. It might not be able to tell the small difference between phases, such that it cannot assign very delicate weights for each feature, and chooses to play safe by always using end-systole frames and stably producing an acceptable fused feature rather than finding the best possible weighting. Fourth, the important phase(s) for each view is different, such that the model might have failed to find a global relativity that satisfies both the relative view importance and phase importance. Namely, the model focuses mostly on ensuring that at each phase, the raw weights satisfy SAX > RVLA > 4CH > 2CH, and focuses less on a delicate relative phase-weight ranking, because preferring SAX components can already lead to a good performance.

Previously reported methods are often limited to either single-view input [10], [11], [12], [13], [14], predefined features [10], [11], [12], [13], requiring image segmentation [10], [11], [12], [13], or performing only few-class classification [10], [11], [14], [15], [16], [17]. In comparison, our approach alleviates the need for segmentation, regresses mPAP directly instead of binary classifying subjects, is capable of fusing cine series from multiple views, and provides certain level of model explainability. In other scenarios where fusing cine loops from multiple views is important for deriving a prediction, the method might be applicable. An important extension of the work would be applying the method to analyze ultrasound cine loops, since ultrasound suits well with the scenario of deriving a preliminary diagnosis quickly, easily and at low cost, obviating the need for RHC. Additionally, other benefits of the model could be investigated when applied to ultrasound cine loops. The design gives it the potential to scale to a large number of input views without having to increase the parameter size. It can also be trained and inferenced on acquisitions with missing, repeated, and unlabeled views. Further evaluation of this could therefore be beneficial.

## 5 Limitations

Before the method can be applied clinically, there are limitations to overcome. First, the current model is trained on a dataset that consists of mainly suspected PH subjects. If the future use case is different, for example, fast screening for PH in a general population, then the model will need fine-tuning on a dataset that is similar to the population of application. Second, although the current training cohort includes a diversity of subjects, the method is not yet validated on an external cohort acquired at different medical centers with different MR scanners. External validation would be important for verifying the generalization ability of the model. Third, in this work, we investigated the pixel importance at the chamber level. A more detailed investigation of the informative value of specific anatomical features, such as size, shape, or signal intensity, would be an interesting topic for future research. Finally, in this study, we tested only predicting mPAP, PAWP, and PVR, but predicting more hemodynamic parameters might be necessary in the future to provide more detailed information.

## 6 Conclusion

We have demonstrated the feasibility of estimating hemodynamic parameters non-invasively with a CNN, using MR cine series from four views, revealing key contributing features at the same time.

## Funding

This study/research is supported by the National Institute for Health and Care Research (NIHR) Sheffield Biomedical Research Center (NIHR203321). The research was also supported by NIHR AI in Health and Care award (grant no. AI_AWARD01706) The views expressed are those of the author(s) and not necessarily those of the NIHR or the Department of Health and Social Care.

## Author contributions

Xiaowu Sun: Writing—review and editing, Methodology. Li-Hsin Cheng: Writing—review and editing, Writing—original draft, Methodology, Formal analysis, Conceptualization. Robin Condliffe: Writing—review and editing, Data curation. Charlie Elliot: Writing—review and editing, Data curation. Samer Alabed: Writing—review and editing, Data curation. David G. Kiely: Writing—review and editing, Data curation. Rob J. van der Geest: Writing—review and editing, Supervision, Project administration, Funding acquisition, Data curation, Conceptualization. Andrew J. Swift: Writing—review and editing, Data curation, Conceptualization.

## Ethics approval and consent

Ethical approval for the study was granted by the local ethics committee and institutional review board (ASPIRE, reference c06/Q2308/8; REC 17/YH/0016).

## Declaration of competing interests

The authors declare that they have no known competing financial interests or personal relationships that could have appeared to influence the work reported in this paper.

## Data Availability

The materials underlying this article can be shared upon reasonable request from the corresponding authors.
