# Supplementary material for: Mean pulmonary artery pressure prediction with explainable multi-view cardiovascular magnetic resonance cine series deep learning model
Source: J Cardiovasc Magn Reson. 2024 Dec 5;27(1):101133. doi: 10.1016/j.jocmr.2024.101133 (PMC11782807; doi:10.1016/j.jocmr.2024.101133)
Supplement: Supplementary file 1 — Supplementary material [file mmc1.docx]

**Supplementary Materials**

**A. Model design and supervision**

The model was designed in this specific way for several reasons: (1) Providing explainability. (2) Being scalable to a large number of views, such that in the future, MR and ultrasound images from much more different views can be incorporated. (3) Being adaptable to an arbitrary number of input views, as well as repeated views, missing views, or unlabeled views that often occur in ultrasound.

Both the 80 initial frame features, the 24 intermediate features, and the final feature can undergo the shared Regression Layer to generate a regression prediction, since they all have the same dimension. As a result, all of them can be supervised. In fact, since the AFFB accepts an arbitrary number of inputs, any random combination of the original 80 frames can be fused into an intermediate feature and be supervised to predict the target. Here, other than supervising the final feature, we choose to supervise also the intermediate features, such that the spatial features for each phase and the temporal features for each view are also trained to predict the target as accurately as possible. The loss function is as Equation (S1),

$L= \alpha\cdot L_{main}+\beta\cdot L_{spatial}+\gamma\cdot L_{temporal}$ (S1)

where $\alpha, \beta, \gamma$ are chosen empirically to be 0.9, 0.05, and 0.05, respectively. Both $L_{main}$, $L_{anatomy}$, and $L_{motion}$ are Mean Square Error (MSE) losses, comparing predictions derived from features and the ground truth mPAP measured by RHC, where $L_{main}$ is the MSE of the final feature, $L_{spatial}$ is the averaged MSE of the intermediate spatial features, and $L_{temporal}$ is the averaged MSE of the intermediate temporal features.

**B. Attention weights**

Figure 5(b, c) presents the raw attention weights, which allows comparing the relative importance of features at the same fusion stage altogether. Here, Figure S1 presents the same set of weights but after normalization. It allows visualization of the actual coefficients that were used to weighted-sum the component features in each fusion.

Normalized weights for all view-wise fusions are presented in Figure S1(a). The normalization happens along each vertical gray line in Figure 5(b,c), such that after normalization, in Figure S1(a), all dots along the same vertical gray line sums up to 1. Similarly, normalized weights for all phase-wise fusions are presented in Figure S1(b). The normalization happens along each curve in Figure 5(b,c), such that after normalization, in Figure S1(b), all dots along the same curve sums up to 1.

Note that in Figure 5(b), the raw frame weights are used in both stage-1 view-wise and phase-wise fusion to generate the intermediate features. Recall that the linear layer in AFFB generates a raw attention weight for each component feature. Since it is the same linear layer in the same AFFB shared across all fusions in the whole model, the raw attention weight for a frame feature is the same in stage-1 view-wise fusion and phase-wise fusion. As a result, there is only one raw attention weight per frame feature. The Softmax-normalized weights for a frame feature in view-wise and phase-wise fusion will, however, be different, as shown separately in Figure S1(a, b).

| (a) | (b) |
| --- | --- |
| 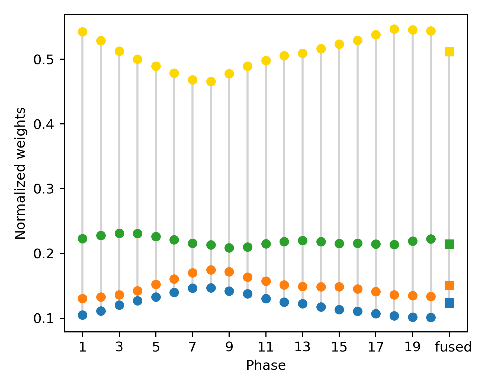 | 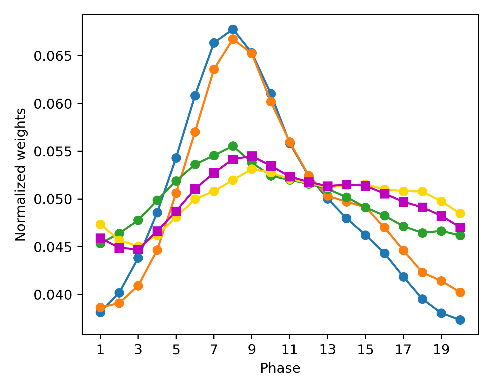 |
| 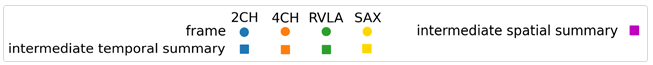 | |

**Figure S1**. Softmax-normalized attention weights. (a) Normalized weights for stage-1 view-wise fusion (round dots), and stage-2 intermediate temporal features (squares). Due to normalization, dots along the same vertical gray line sum up to 1. (b) Normalized weights for stage-1 phase-wise fusion (round dots), and stage-2 intermediate spatial features (squares). Due to normalization, dots along the same curve sum up to one.
